# Expansion microscopy reveals neural circuit organization in genetic animal models

**DOI:** 10.1117/1.NPh.12.1.010601

**Published:** 2024-12-20

**Authors:** Shakila Behzadi, Jacquelin Ho, Zainab Tanvir, Gal Haspel, Limor Freifeld, Kristen E. Severi

**Affiliations:** a New Jersey Institute of Technology, Federated Department of Biological Sciences, Newark, New Jersey, United States; b Albert Einstein College of Medicine, Departments of Genetics and Neuroscience, Bronx, New York, United States; c Rutgers University, Departments of Cell Biology and Neuroscience and of Genetics, New Brunswick, New Jersey, United States; d Technion - Israel Institute of Technology, Faculty of Biomedical Engineering, Haifa, Israel

**Keywords:** expansion microscopy, resolution, neural circuits, connectomics, genetic models

## Abstract

Expansion microscopy is a super-resolution technique in which physically enlarging the samples in an isotropic manner increases inter-molecular distances such that nano-scale structures can be resolved using light microscopy. This is particularly useful in neuroscience as many important structures are smaller than the diffraction limit. Since its invention in 2015, a variety of expansion microscopy protocols have been generated and applied to advance knowledge in many prominent organisms in neuroscience, including zebrafish, mice, *Drosophila*, and *Caenorhabditis elegans*. We review the last decade of expansion microscopy–enabled advances with a focus on neuroscience.

## 1 Introduction

Until recently, important neuronal structures have been too small to resolve with light microscopy. Resolution in microscopy is the capacity to distinguish closely spaced objects as separate entities.^1^ The resolution of light microscopy is physically limited by the wavelength of light and the collecting angle of the front lens to ∼250  nm, known as the “diffraction limit.”^2^^,^^3^ Many neuroanatomical structures, such as chemical synapses, gap junctions, and the smallest of neurites, are smaller than the diffraction limit. The two solutions for this issue have been to use either electron microscopy, in which the diffraction limit is dictated by a much smaller wavelength that allows molecular level resolution, or innovative modifications of optics and image analysis in a suite of approaches known as super-resolution light microscopy.^4^^–^^6^ Expansion microscopy (ExM) is a recent super-resolution microscopy technique that offers practical and scientific advantages.^7^ The four primary advantages of ExM are: (1) nano-scale resolution using standard, accessible, and broadly available diffraction-limited microscopes; (2) rapid imaging and processing time in comparison to electron microscopy and most other super-resolution methods; (3) relatively low cost; and (4) an ability to image large tissue samples at resolutions beyond the diffraction limit.

ExM increases the resolution of standard fluorescent microscopes beyond the diffraction limit by physically expanding the sample. The samples are embedded in polyelectrolyte hydrogels that isotropically expand upon the addition of pure water. Once expanded, features smaller than the diffraction limit may become resolvable using standard diffraction-limited microscopes. Thus, the method is compatible with existing laboratory workflows.

By changing the sample rather than the optics or illumination protocol, the ExM approach differs from that of other super-resolution methods, including stimulated emission depletion (STED) microscopy, structured illumination microscopy (SIM), photoactivated localization microscopy (PALM), and stochastic optical reconstruction microscopy (STORM).^8^ A limitation these techniques have in common is that they can only be used with thin sections.^9^ STED requires a precisely aligned dual-laser setup: one laser excites a focal area, whereas a second laser selectively depletes fluorescence in a toroid region around the focal point to achieve super-resolution.^10^ SIM enhances resolution by projecting patterned light onto the sample for computational reconstruction. This method is limited to doubling the optical resolution, and it requires sophisticated light systems and precise control over illumination patterns.^11^ PALM and STORM both rely on the stochastic activation and precise localization of individual fluorescent molecules to build high-resolution images over time.^12^^,^^13^ PALM uses photoactivatable fluorescent proteins, whereas STORM typically uses photoswitchable dyes. Both methods require sensitive detection equipment, such as high-performance cameras, and advanced computational algorithms for image reconstruction.^14^ In addition, they require long acquisition times as each imaged plane must be imaged multiple times to allow the reconstruction of the structural information from single-particle localization data. Although all these techniques achieve resolutions below the diffraction limit, they require significant investment in specialized hardware and software and limit the sample size that can be imaged.

ExM, by contrast, offers a more accessible and scalable approach, leveraging broadly available microscopy equipment to study large biological specimens with high resolution. The enhancement of resolution is uniform along all three imaging planes, and the sample processing results in an entirely clear sample, which obviates the need for additional clearing techniques. The sample preparation for ExM can often be simply incorporated into existing workflows with the addition of a few days of processing time in exchange for dramatically improved imaging results. However, ExM requires fixed samples, whereas other methods mentioned above can be compatible with live super-resolution microscopy.

ExM permits mechanistic insights at multiple levels of organization, from ultrastructure to tissue, such as the organization of proteins within and between gap junctions and chemical synapses. Here, we review how ExM has been applied to reveal previously unknown features of neural circuits in model animals used in neuroscience research.

### 1.1 Expansion Microscopy

The central feature of ExM is the isotropic expansion of tissues that have been embedded in a polyelectrolyte gel. The gel expands equally in each dimension, retaining spatial relationships between features in the original tissue. Importantly, it is possible to choose which molecules will be anchored to the gel (e.g., proteins, nucleic acids, or lipids), and thus, different types of cellular structures can be captured. The standard protocol attaches the amine moieties of proteins to the gel (see proExM below). ExM typically expands a sample by about fourfold,^7^^,^^15^ but the appropriate optimization of the gel composition can yield tenfold single-step expansion,^16^^,^^17^ and iterative expansion approaches can be used to achieve expansions of about twentyfold.^18^^,^^19^ A recent protocol achieves 20× single-step expansion through further optimizing gel composition and the polymerization environment.^20^ Samples imaged with ExM can be stained with fluorescent antibodies both before or after the expansion step.^21^

**Fig. 1:**
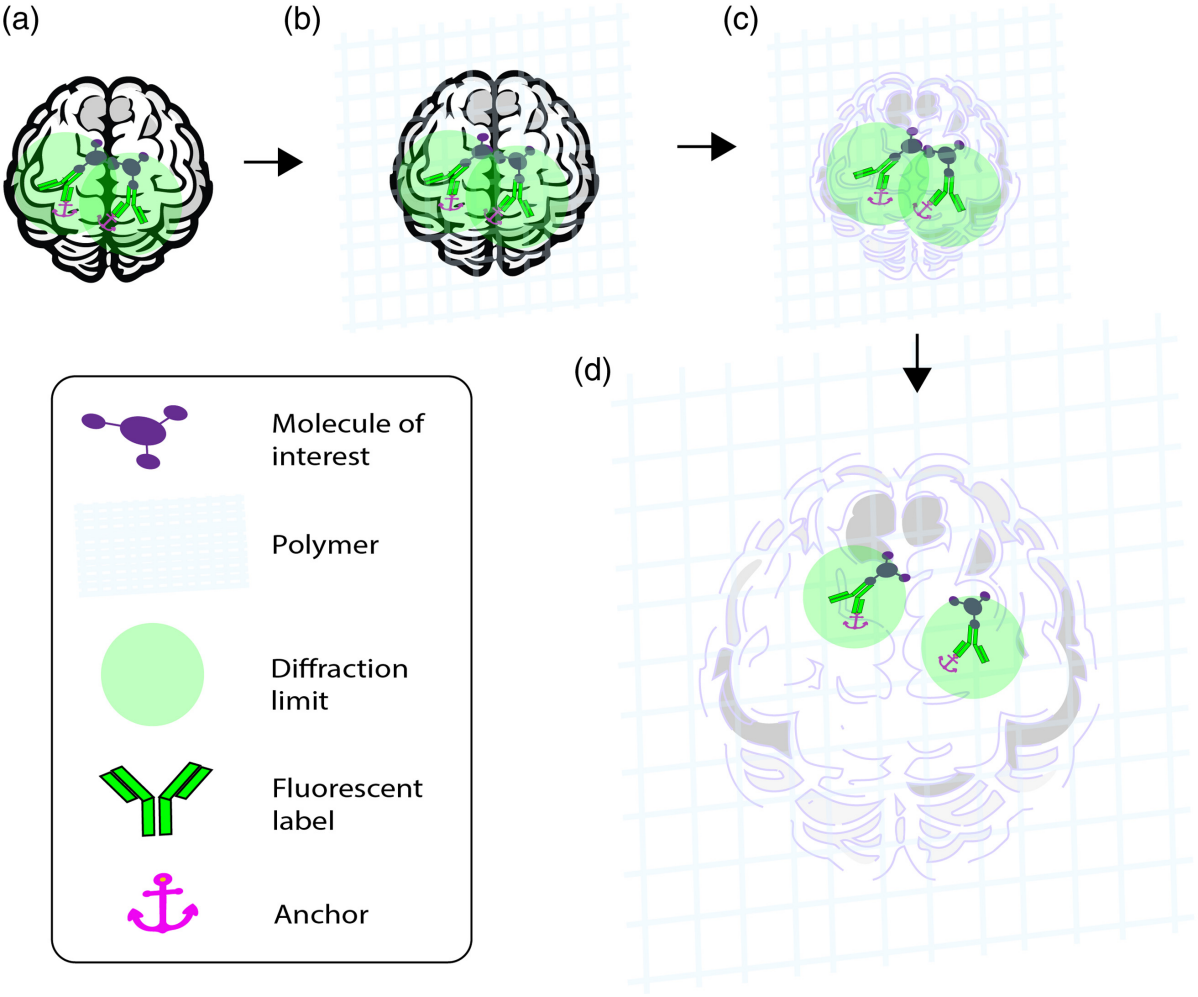
Major steps of an expansion microscopy protocol. (a) Anchoring biomolecules within the sample to gel elements. (b) Gelation of the sample and forming the hydrogel polymer. (c) Digestion of the sample through proteolysis. (d) Expansion of the hydrogel with water, enhancing resolution by spatial separation of the fluorescent labels, surpassing the diffraction limit of ∼250 nanometers for light microscopy.

Isotropic expansion via hydration of a sample embedded in a hydrogel involves four major steps (Fig. 1).^22^ The first step is anchoring, in which the sample is incubated in a solution containing an anchoring agent [Fig. 1(a)]. The anchoring step attaches the sample to the polymerizing hydrogel. A key innovation is the use of acryloyl-X (AcX) as an anchoring agent. Incubation of the sample in AcX enables the crosslinking of amines in proteins and other molecules to acrylamide monomers in the gel. As a result, native proteins are retained—this version of ExM is known as protein-retention ExM (proExM).^15^ The second step is gelation, in which the sample is bathed in monomers (sodium acrylate and acrylamide) and a cross-linking agent that first diffuse into the sample at a low temperature and are then allowed to polymerize to form the gel [Fig. 1(b)]. The third step is digestion, in which the sample is chemically homogenized via the application of proteolytics and detergents that disrupt the chemical bonds of molecules in the sample [Fig. 1(c)]. Homogenization is necessary to avoid distortion of the sample during the expansion phase, whereas the anchoring preserves the relative locations of fluorophores that mark the structures of interest within the sample. Finally, the sample is expanded by placing it in water [Fig. 1(d)].

### 1.2 Tissue Preparation

#### 1.2.1 Anchoring links sample components to the hydrogel monomers

Currently, most ExM protocols rely on AcX as an anchor,^15^ which enables crosslinking of amine moieties in proteins and other molecules to acrylamide monomers in the gel. Anchoring with AcX is compatible with conventional fluorescent labeling techniques, including immunohistochemistry. Importantly, this method is compatible with not only genetically expressed fluorescent proteins and commercially available antibodies, but also nucleic acids incubated with an additional anchoring agent such as Label-IT, which covalently attaches amines to nucleic acids.^23^ We note, however, that some fluorescence loss can occur in the digestion and polymerization steps and the extent to which signal is lost depends on the fluorophore chemistry. In particular, cyanine-based dyes (e.g., Alexa 647, cy2, cy3, and cy5) are associated with significant signal loss in proExM and thus are not recommended.^15^ *N*-acryloxysuccinimide (AX) has recently proven to be an anchoring agent similar to AcX and is available at a much-reduced cost.^20^

#### 1.2.2 Gelation polymerizes and solidifies the hydrogel in which the sample is embedded

Gelation results in a 3D matrix of crosslinked polymers to which molecules of interest in the sample are chemically anchored. The gelation process occurs in a chamber that holds the sample and the solutions that form the gel. The chamber must be large enough to cover the sample in its entirety and establish the shape of the gel. The chamber is then sealed with a coverslip and incubated to allow polymerization.^22^ The size and shape of the chamber are additional critical features that determine the user’s ability to manipulate the gel and its structural integrity during processing. After the monomer solution diffuses throughout the sample in a 4°C environment, a change to 37°C triggers the polymerization of the sodium acrylate and acrylamide monomers.^24^ The crosslinking polymers form a dense matrix, and the anchoring agent forms covalent bonds between amines in the sample and acrylamide in the gel.

Most existing protocols for ExM depend on bis-acrylamide, which cross-links acrylamide monomers in the gelation step. N,N-dimethylacrylamide (DMAA) is a self-polymerizing agent, which forms more structurally robust hydrogels that are able to withstand 10× or even 20× expansion.^20^^,^^25^ However, DMAA is even more sensitive to atmospheric oxygen, which reacts with intermediate radicals and interferes with radical-dependent gel cross-linking. Nitrogen gas can be used to flush out ambient oxygen from a working environment for uniform gelation.^20^

#### 1.2.3 Digestion homogenizes the sample through proteolysis to minimize the distortion of the sample during later expansion

Digestion is typically performed using proteinase-K.^15^ For experiments in which labeling is performed after the expansion step, this step can be replaced with autoclaving the samples in detergents such as SDS and Triton, or via the use of a milder protease, to preserve epitopes while denaturing protein aggregates to allow gel expansion.^21^

#### 1.2.4 The sample-embedded hydrogel absorbs water and expands

Water is attracted to the polarized ends of the hydrogel molecules and forms dipoles around the negative ionic charges in the gel mesh, causing the polymer chain molecules to extend and the gel to swell isometrically.^26^ Samples typically expand approximately fourfold (although see additional variants described below).

### 1.3 Expansion Variations

#### 1.3.1 High expansion factors and resolution

**Fig. 2:**
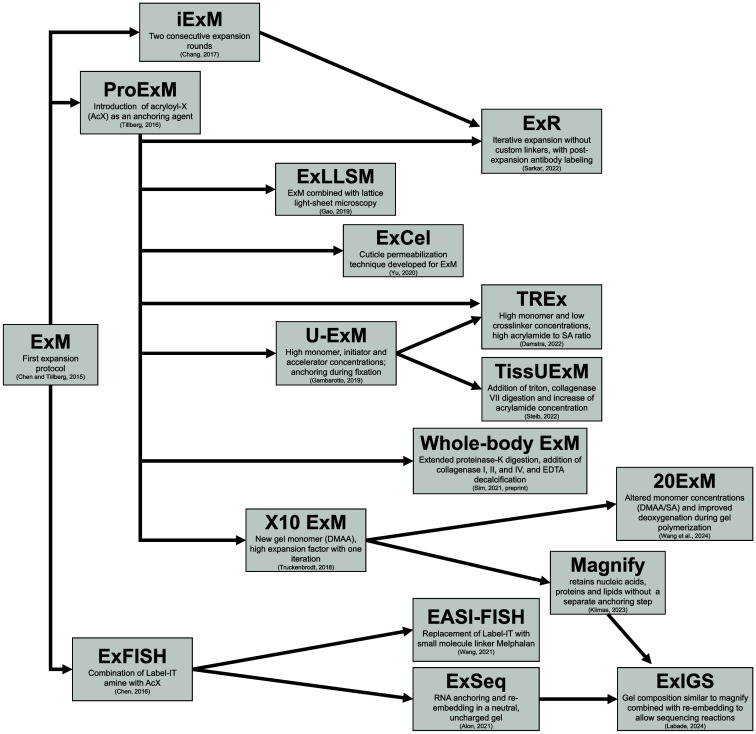
Variations of Expansion Microscopy. ExM: In the original protocol, the monomer gel solution includes eight components—sodium acrylate; acrylamide; MBAA, *NN*′-methylenebisacrylamide; PBS, phosphate-buffered saline; APS, ammonium persulfate; TEMED, $N,N,N^{\prime },N^{\prime }$-tetramethylethylenediamine; 4HT, 4-hydroxy-tempo. Sodium acrylate and acrylamide form the polymer as they are cross-linked by MBAA. 4HT is a polymerization inhibitor that prevents premature gelation before the monomer solution diffuses throughout the sample tissue; TEMED serves as an accelerator; APS serves as the initiator and thus is added last to the solution.^7^^,^^27^ ProExM: Acryloyl-X is added as an anchoring agent that binds proteins to the polymer.^15^ iExM: An iterative approach to ExM, where the initial polymer is broken down, and a second swellable polymer mesh is formed in the space newly opened by the first expansion. This was implemented using custom-made DNA oligonucleotides conjugated to secondary antibodies and polymer anchoring moieties,^18^ such that only the specifically labeled structures of interest are attached to the polymer. ExR: AcX was applied in iExM for broad anchoring of proteins and antibodies to the polymer before post-expansion staining.^19^ 10× expansion: A higher expansion factor was obtained by replacing acrylamide with N,N-dimethylacrylamide acid (DMAA) as the main monomer together with sodium acrylate.^17^ 20ExM: A single-step twentyfold expansion protocol without iterative steps, using optimized hydrogel integrity and oxygen-controlled gelation.^20^ TREx: By decreasing the crosslinker and monomer concentrations, the hydrogel maintains structural integrity and increases the expansion factor tenfold in a single gelation step.^16^ U-ExM: For expanding cultured cells and analyzing proteins at higher resolution. Anchors proteins to the polymer by applying acrylamide and formaldehyde rather than using AcX.^28^ iU-ExM: Enables high-resolution visualization of cellular structures by combining ultrastructure expansion with iterative expansion, achieving near-SMLM resolution for detailed molecular imaging in tissues and organelles.^29^ TissUExM: To penetrate thicker tissue, this protocol adds triton to the monomer solution and increases the concentration of acrylamide. In addition, a collagenase VII digestion step is introduced after gelation, followed by a denaturation step and post-expansion staining.^30^^,^^31^ Whole-body ExM: to homogenize and expand an entire zebrafish with multiple tissue types such as bones, cartilage, muscles, and soft tissues, this protocol extended the proteinase-K digestion time, added collagenase I, II, IV digestion, and decalcification with ethylenediaminetetraacetic acid (EDTA).^32^ ExFISH: Preserves the integrity and spatial positions of RNA molecules in cells and tissue slices. The RNA is stained by fluorescence *in situ* hybridization (RNA-FISH) amplified with hybridization chain reactions (HCRs) that enable the resolving of single RNA molecules. Multiple rounds of FISH increase the number of target RNA sequences.^23^ EASI-FISH: Replaces Label-IT in the ExFISH protocol with a smaller molecule linker, melphalan, to retain and anchor nucleic acids to the ExM polymer. This linker is cost-effective, improves signal-to-noise ratio, and optimizes reagent penetration.^33^ ExLLSM: Addresses microscopy working distance that limits sample thickness with a light sheet microscopy pipeline.^34^^,^^35^ ExCel: Adapted ExM to the nematode *C. elegans* by adding steps for cuticle permeabilization.^36^^,^^37^ Magnify: Achieves higher expansion factors and enhanced imaging resolution by adding N,N-dimethylacrylamide (DMAA) to the monomer solution alongside acrylamide and sodium acrylate. This protocol combines the high expansion factor of 10× expansion with protein retention strategies from ProExM, allowing for detailed imaging of proteins in large or thick samples.^25^ ExSeq: Enables spatially resolved RNA sequencing by anchoring RNA molecules to the gel using reagents such as LabelX and maintaining a neutral pH during expansion to preserve RNA integrity. The protocol involves re-embedding the expanded gel in a non-expandable gel to facilitate sequencing procedures.^38^ ExIGS: Facilitates in-gel sequencing of DNA or RNA molecules with higher expansion factors by using a modified polyacrylate gel incorporating DMAA and Tn5 transposase. By adding rolling circle amplification (RCA) and sequencing-by-synthesis (SBS) buffers with increased gel porosity, it supports the enzymatic diffusion necessary for sequencing. The protocol includes re-embedding the expanded gel in a non-expandable gel for sequencing procedures and is derived from Magnify and ExSeq.^39^

One approach to obtain expansion factors higher than approximately fourfold, and thus higher spatial resolution, is the iterative application of the expansion process. Iterative expansion (iExM) involves two consecutive expansion rounds. In iExM (Fig. 2), an expansion factor of ∼20-fold is obtained, yielding a resolution of 20 to 25 nm with standard confocal microscopy imaging.^17^

In an alternative approach, 10× expansion and TREx (Fig. 2), 10-fold expansion is achieved in a single step via optimization of the gel recipe.^16^^,^^17^ The most recent improvement of this permits 20× single-shot expansion,^20^ by developing a super-absorbent hydrogel recipe, tightly controlling oxygen levels during gelation and optimizing the gelation times according to the biological sample type (Fig. 2).

#### 1.3.2 Compatibility with light microscopes: tradeoffs and considerations

When working with large, expanded samples, the working distance between the imaged plane and the microscope objective becomes an important consideration. Working distance depends on the sample, expansion factor, and the microscope. Water-dipping objectives and upright microscopes offer the best working distances and are thus advantageous over inverted microscopes and oil or water immersion objectives. However, inverted microscopes are in more common use, and the latter objectives are associated with higher numerical apertures. Superficial target molecules within the sample may be accessible with objectives that have working distances smaller than the gel thickness. Consequently, the orientation of the sample within the gel and the orientation of the gel are important when imaging. Moreover, it is important to fit the chamber height as precisely as possible to the sample depth to minimize empty space.^22^

#### 1.3.3 Labeling target proteins and nucleic acids

To pinpoint spatial relationships among molecular structures, proteins and nucleic acids are tagged with fluorescent molecules or other markers that have high specificity to the molecules under study. ExM is typically paired with protein and nucleic acid labeling techniques, such as immunohistochemistry and *in situ* hybridization, to tag molecules of interest. By anchoring the molecules of interest to the gel using AcX for proteins, or label-IT together with AcX for nucleic acids, and then expanding the sample, ExM enhances the detection of labeled proteins and nucleic acids, making it easier to study complex and densely packed structures within cells and tissues.^15^^,^^20^^,^^23^^,^^40^

#### 1.3.4 Pre- versus post-expansion labeling

Molecules of interest can be labeled either before or after expansion treatment depending on the scientific question and preferred imaging tool. Pre-expansion labeling is most common in established protocols and procedures; fluorophores are often better able to withstand the digestion step necessary to soften and homogenize samples for expansion, whereas native fluorescent proteins may be proteolyzed and unfit for labeling post-expansion. However, post-expansion labeling is ideal for densely packed targets that benefit from “decrowding,” an increase in target accessibility that has helped visualize nanostructures such as amyloid plaques of Alzheimer’s disease model mice samples.^19^ In specific cases, as the expansion process physically separates proteins, it can render previously inaccessible targets accessible for antibody labeling, as native inter-protein distances can be shorter than the size of antibodies.^19^^,^^20^ Moreover, it can allow for more precise localization of molecules of interest by reducing the linkage error, an error in spatial visualization measured by the distance between the molecule of interest and the fluorescent reporter due to the size of antibodies used as reporters;^41^ the relative size of the antibody with respect to the structure of interest becomes significantly smaller when the antibody is applied to the expanded sample. In addition, post-expansion labeling avoids fluorescence signal loss during homogenization and polymerization, which can be significant.^42^ Although post-expansion labeling allows for flexibility in experimental design and has greater signal amplification, it requires a gentler digestion step to retain target isotopes and greater reagent quantities and thus costs more than pre-expansion labeling due to the increased size of the sample. To mitigate the signal loss during the expansion step, combining pre- and post-expansion labeling can further amplify the labeling signal.^20^^,^^36^

#### 1.3.5 Limitations of ExM

Several technical challenges remain in ExM, which future advancements aim to address. One such challenge is consistency in protocol application and obtained expansion factors between samples of the same type and to a greater extent in different types of samples. Moreover, ExM protocols, particularly ones associated with high expansion factors, can involve complex steps and require adaptation for and validation in new types of samples. Nevertheless, recent developments in automated sample preparation and streamlined protocols have shown promise in reducing benchwork time and improving workflow efficiency.

Additional challenges arise from the size of the expanded samples, particularly when expansion factors are 10× or higher. This is a challenge for the imaging itself, as some microscope imaging chambers have limited sizes, high NA objectives have limited working distances and thus are ill-suited for imaging these samples—giving rise to a resolution limit (albeit this limit is far more than compensated by the expansion factor), and large samples require the use of large working distance objectives that poorly fit, e.g., inverted microscopes. Moreover, sample volumes can increase by three orders of magnitude, which can make imaging times very long even on fast microscopes. Light-sheet and upright spinning disk confocal microscopes are thus best suited for imaging expanded samples. Large sample volumes also dilute the fluorescent signal, requiring signal amplification or the use of post-expansion staining, which requires very large amounts of antibodies (see pre- versus post-expansion labeling). Finally, as ExM achieves higher resolutions and sample volumes increase, the size of resulting datasets grows substantially, increasing storage and computational demands and requiring the development of appropriate software solutions for data analysis.^34^

## 2 Expansion Microscopy Across Species

Expansion microscopy has been applied to many commonly used model systems, including zebrafish (*Danio rerio*), mice (*Mus musculus.),* fruit flies (*Drosophila melanogaster*), and nematode worms (*Caenorhabditis elegans*). ExM enhances microscopic details while preserving the overall spatial structure, which is particularly useful for understanding neural structures. In zebrafish, proExM has detailed synaptic connections.^15^ In mice, ExFISH has mapped deep brain regions.^23^ For *Drosophila*, ExLLSM has improved imaging speed and clarity.^34^ In *C. elegans*, ExM has revealed protein arrangements and centriole dynamics.^43^ In each of these systems, several ExM variations have been used to answer fundamental questions about neuroanatomical organization at the subcellular level.

### 2.1 Macroscopic to Ultrastructural Resolution in Zebrafish

Zebrafish (*Danio rerio*) are widely used in biomedical research partly due to their genetic accessibility, small size, and translucent embryos and larvae. Embryos and larvae are typically less than 4 mm in length and are sufficiently transparent that their internal organs can be visualized in intact, living individuals.^44^ This transparency makes zebrafish particularly amenable to *in vivo* optical techniques, including neural activity imaging via genetically encoded calcium indicators using a variety of microscopy approaches,^45^^–^^50^ optogenetic perturbations of neuronal activity, and other optical processes.^51^^–^^54^ Many neural circuits span large areas of the body, and neurons and their processes can be easily visualized throughout the nervous system in young zebrafish. Thus, zebrafish are well-suited for the study of how intact neural circuits give rise to functional behavior. However, the synaptic interfaces among neurons, critical for understanding neuronal circuit function and behavior, are densely packed with proteins that are difficult to visualize using light microscopy. Visualizing and resolving synaptic protein organization below the diffraction limit can increase our understanding of mechanisms of neural circuit function, structure, development, and plasticity.

To understand circuit function, it is advantageous to study simultaneously the macroscopic structures and nanoscopic details. ExM makes this possible by maintaining the overall spatial relationships of cellular-level structures while improving the resolvability of nanoscopic structures. ExM has been used in zebrafish to resolve synaptic connectivity and intra-synaptic protein organization, such as glycine receptors and gap junction organization within densely packed synapses using protein-retention ExM (proExM).^15^^,^^55^^,^^56^ ExM has also been used in zebrafish to resolve structures in large tissue samples such as whole-embryo zebrafish in TissuExM and whole-body expansion.^30^^–^^32^

### 2.2 Resolving Synaptic Connections in Zebrafish

The first demonstration of the utility of ExM in zebrafish came in Freifeld et al.,^56^ where the authors resolved putative synaptic connections between two fluorescently labeled cell populations. This structural information could be highly valuable in complementing functional data from such neuronal populations thought to constitute a neural circuit, e.g., it can reveal how the participating neurons and connectivity patterns vary between individuals to mediate heterogeneity in behavior or how they change in learning and development.^56^ One such circuit in zebrafish, which has been a focus of research for decades, is the escape circuit. Like most fish and amphibian species, zebrafish have a pair of large command neurons for escaping predation, the Mauthner cells.^57^ The subcellular spatial distribution of electrical and chemical synaptic inputs onto Mauthner cells, which ultimately determine the initiation of escape behavior, is below the diffraction limit.^58^^–^^60^ Several studies using ExM have resolved organizational aspects of synaptic structure in Mauthner cell synapses and revealed insights into the mechanisms of Mauthner cell function.

Glycinergic inhibition onto the Mauthner cells is an essential gating mechanism controlling their activity and thus modulating escape response thresholds and directionality.^61^ Researchers have used proExM to image larval zebrafish brains and resolve protein organization within synapses on the Mauthner cells. In particular, glycine receptors in glycinergic synapses on the Mauthner cells were found to often form annuli, and the density of synaptic proteins in axon-cap synapses was found to be heterogeneous.^56^ The annular organization of glycine receptors on larval zebrafish Mauthner cells was consistent with the identified structure of glycinergic synapses in other examples, including mammals.^62^^–^^64^ This work paves the way to reveal how this organization is functionally modulated during synaptic plasticity. ProExM can be used to answer questions that functionally link nano-scale synaptic plasticity to circuit function and behavior as the structure can be captured subsequently to functional experiments in this animal model.

In another study, researchers mapped the distribution of proteins at a single synaptic input to the Mauthner cell to reveal the structural support provided by the molecular scaffold underlying gap junctions.^55^ Cárdenas-García et al.^55^ found multiple gap junctions and components of adherens junctions occupying most of the synaptic area, suggesting a functional association between these structures. These synapses are known to be mixed electro-chemical synapses. However, glutamate receptors were confined to peripheral portions, indicating that the majority of the synaptic area functions as an electrical synapse.^55^ By identifying gap junctions at a single synapse level, electric synapses on the M-cell can be used as a model to study plasticity and protein-specific synapse configurations with functional implications. Moreover, ExM can shed light on how chemical and electrical synapses are co-modulated by experience to mediate learning.

ExM was also used to resolve the spatial relations between neural fibers and glial processes.^65^ This is built upon a previous demonstration of ExM-enhanced tracing of radial glial cells in visual processing areas.^56^ As glia are important for synaptic function and plasticity, resolving such spatial relations can lead to key insights into the underlying mechanisms of neural-glia interactions and glial effects on neural circuit functions and behavior.

### 2.3 Resolving Structures Within Whole Intact Zebrafish with ExM Variants

In tissue ultrastructure expansion microscopy (TissUExM) and whole-body ExM (Fig. 2), protocol advancements, and in particular added steps for digestion of collagen and bones, allowed the expansion of intact larval zebrafish, zebrafish embryos, Drosophila wing, and mouse embryo.^30^^–^^32^ Overall, TissUExM was designed to ensure the isotropic fourfold expansion of heterogeneous tissue samples. TissUExM revealed endogenous proteins in the brain and spinal cord of whole zebrafish embryos to analyze cilia heterogeneity and tissue-specific defects associated with ciliopathies in multiple body locations simultaneously.^30^ These locations included the olfactory bulb and longitudinally distributed structures such as the hair cells of the lateral line and cilia of the spinal cord. This approach permits the comparison of ultrastructural details in spatially distinct tissues within the same intact embryo.

Whole-body ExM (Fig. 2) was successfully applied to larger, denser, and older animals, such as larval zebrafish up to 8 days post-fertilization and even juvenile fish (several months old). It enabled an increase in resolution from ∼1  μm to 60 nm.^32^

Both TissUExM and whole-body ExM can achieve the expansion of intact, and several-millimeter-long animals are compatible with simultaneous immunohistological staining and with genetically encoded fluorescent protein labeling. Such techniques are conducive to the observation of intricate neural connections and signaling pathways throughout the nervous system from the brain to the spinal cord and allow for the exploration of various neural circuits involving complex spatially distributed signals and connections that vary along the neuraxis. These ExM techniques provide an essential link between structure and function, revealing not only subsynaptic structures but also intricate neural connections and signaling pathways throughout the nervous system from the brain to the spinal cord.

### 2.4 Unpacking Synaptic Connectivity in Thick Tissue of Mice

Mice (*Mus musculus*) are biomedical research’s most widely used animal species. Because of the extensive literature on mice and well-established protocols for genetic manipulation, mice are used to address experimental questions on the relationship between genes and neural circuits and to test models of neurological and psychological diseases.^66^ However, mice share the same resolution problem as other model systems where sub-cellular structures are below the diffraction limit. Furthermore, mouse brains are substantially larger than some non-mammalian species such as zebrafish, and as a result, the brain has to be sectioned and reconstructed to allow light penetration for imaging. Imaging mouse tissue remains challenging, despite recent advances in tissue clearing.^67^^,^^68^ Fortunately, ExM also clears the sample in the digestion step, homogenizing the refractive index throughout the sample and limiting the scattering of photons within the sample.^7^ Below, we describe how anatomical regions deep in the mouse brain were studied with ExM and iterative RNA-FISH of brain slices. ExFISH combines molecular information, enabling the identification of cell types and sub-regions, with the resolving of fine neural processes and structures.^33^ This advance was predicated upon the ability to combine RNA-FISH and ExM, pioneered as ExFISH.^23^ The RNA is stained by fluorescence *in situ* hybridization (RNA-FISH) amplified with hybridization chain reactions (HCRs) that enable resolving single RNA molecules. Multiple rounds of FISH increase the number of target RNA sequences.^23^ In an elegant example of decrowding to understand nanostructure colocalization, expansion revealing (ExR) shows the distributed pathological nano-markers of Alzheimer’s disease.^19^ These recent advances have opened new avenues of investigation to observe RNA and tightly clustered proteins deep in the mouse brain, which were previously inaccessible. Magnify builds on the 10× and proExM protocols to enable nanoscale imaging across diverse biological samples, achieving resolutions as fine as 25 nm.^25^ By preserving a broad range of biomolecules—including nucleic acids, proteins, and lipids—Magnify serves as the foundation for advanced protocols such as ExIGS, a genomic DNA sequencing method.^39^

Expanding on these capabilities, methods such as expansion sequencing (ExSeq) and expansion *in situ* genome sequencing (ExIGS) offer nanoscale resolution for *in situ* RNA and genome sequencing, respectively. ExSeq allows untargeted transcriptomics in complex tissues, such as tumor microenvironments and neuron-dense areas, whereas ExIGS links nuclear abnormalities to chromatin repression hotspots, illuminating how structural changes affect gene regulation, especially relevant in aging and disease research.^38^^,^^39^

#### 2.4.1 Spatial transcriptomics with ExM

Expansion fluorescence *in situ* hybridization (ExFISH) (Fig. 2) was the first ExM variant that introduced the anchoring of nucleic acids, such as RNA, to the gel.^23^ Expansion-assisted iterative fluorescence *in situ* hybridization (EASI-FISH) (Fig. 2) improves spatial precision of *in situ* hybridization in thick tissue specimens via protocol optimization and the use of a distinct anchoring molecule.^33^ EASI-FISH was used to spatially determine transcripts from many genes simultaneously across a 300-μm-thick slice and identified previously unknown morphological diversity in deep regions such as the lateral hypothalamic area of mice. Using EASI-FISH in combination with genetic data from single-cell RNA-Sequencing (scRNA-Seq), researchers characterized marker genes in the lateral hypothalamic area and classified nine spatio-molecular regions.^33^

#### 2.4.2 Revealing hidden protein structures with ExM

In expansion revealing (ExR) (Fig. 2), proteins are “decrowded” by two iterative expansion steps such that the increased distance between proteins allows post-expansion applied antibodies, larger than the space between these tightly packed proteins, to access their targets. Decrowding allows the labeling of targets, which cannot be visualized well even using super-resolution techniques, as the targets are challenging to label precisely until they are expanded. Using ExR, researchers revealed the nanoscale coordination of presynaptic calcium channels with postsynaptic proteins and the periodic nanostructures of amyloid beta plaques in mouse models of Alzheimer’s disease.^19^ This was only possible due to ExR’s capacity to decrowd proteins spatially while imaging with resolution on par with super-resolution techniques.

### 2.5 Looking Across the Entire Brain with Unparalleled Resolution in *Drosophila*

The fruit fly (*Drosophila melanogaster*) is commonly used in neurobiological research due to its practical advantages, available genetic tools, and well-documented connectome.^69^ Flies are easy and inexpensive to maintain, have a short life cycle, and produce large numbers of offspring. *Drosophila* were first used to understand genetic inheritance, chromosome mapping, and genetic mutations.^70^ They share many conserved genes with humans, including those involved in cell division, development, and basic body plan formation.^71^ Approximately 75% of human disease genes have equivalents in flies.^71^ Due to their highly tractable genetics and well-characterized behavioral repertoire, *Drosophila* can be used to identify neuronal circuits and genes underlying behavior.^72^

A complete synaptic-resolution connectome of the *Drosophila* larval brain^73^ and an adult brain connectome^74^ were published, based on datasets from electron microscopy. However, impressive projects on this scale with traditional imaging methods can be expensive and slow. These atlases require expensive and not broadly available equipment, significant computation time, and resources and often require large cohorts of research groups for their generation. In contrast, ExM can be applied within days in most equipped laboratories and makes it possible to collect high-resolution information on neural structures in multiple animals. The high-throughput imaging of multiple animals makes it possible to address behavioral heterogeneity and its circuit origins: researchers can compare neural structures in cohorts of animals presenting distinct behaviors, tracking how neural structures and corresponding behaviors evolve during development or are modulated to mediate learning. When ExM was first applied to the imaging of *Drosophila* brains, it was coupled with lattice-light sheet microscopy (LLSM) to obtain enhanced volumetric imaging resolution.^34^^,^^35^

#### 2.5.1 In *Drosophila*, ExM enables the comparison of neural circuit connectivity characteristics between populations

ExM was optimized for application to larval *Drosophila* brains. The presynaptic protein organization observed was consistent with that established with other super-resolution microscopy methods. In addition, ExM allowed more precise quantification of insertion of somatosensory neural dendrites into epithelial cells compared with standard confocal microscopy.^75^

When the expansion was followed by lattice light-sheet microscopy (ExLLSM^34^) (Fig. 2), it allowed for high-resolution imaging of the entire* Drosophila* brain, with the ability to distinguish features that are ∼60  nm apart in the x and y dimensions and 90 nm apart in the *z* dimension. Furthermore, ExLLSM image acquisition was ∼700 times faster than other super-resolution fluorescent microscopy techniques and 1200 times faster than EM.^34^ The technique makes it possible to zoom in on individual synapses and zoom out to see broader patterns in synaptic connectivity, facilitating comprehensive and comparative anatomical investigations.^76^

Using ExLLSM, researchers imaged all dopaminergic neurons across the *Drosophila* brain, visualizing their morphologies in all major brain regions and tracing specific clusters to determine cell types.^34^ They also quantified presynaptic active zones across the brain, providing insights into the local density of synapses and dopaminergic neuron-associated active zones.^34^ ExLLSM has been used to trace axonal branches of olfactory projection neurons and study their bouton arrangements at the calyx and lateral horn across multiple *Drosophila* specimens.^34^

Lillvis et al.^35^ added the development of a high-throughput ExLLSM open-source image analysis pipeline to enhance the accessibility of studying neuronal circuits. Importantly, in this manner, they were able to produce a proof of concept for one of the greatest promises of the application of ExM to neural circuits: due to the high speed and applicability to volumetric samples, ExM can be applied for the comparison of neural circuit connectivity across animal populations. This is a major advance compared with EM connectome generation that provides higher resolution data but for a single animal. Thus, they demonstrated the quantification of synapses in male compared with female fly populations. In the future, this approach can be applied to reveal gender, development, and experience-dependent differences in neural circuits and synapse structure. They also revealed synaptic structural underpinnings of behavior variability in a population. Such questions can only be addressed with rapid, volumetric imaging beyond the diffraction limit.

### 2.6 Localization of Diverse Molecules in *C. elegans*

The roundworm *C. elegans* is the first organism to have complete connectome mapping synapses and gap junctions among neuron types.^77^ Its compact nervous system of 302 neurons is highly stereotyped and capable of encoding essential and interesting behaviors, ranging from reflexive escape from harsh touch^78^ to associative and nonassociative learning.^79^ In addition, because of its small size, with adults measuring around 1 mm long, optical transparency, and genetic tractability, the worm is well-suited for *in vivo* imaging of genetically encoded fluorophores and sensors.^80^^,^^81^

The completeness of *C. elegans*’s community atlases and databases now spans connectomes of multiple developmental time points and of both sexes,^77^^,^^82^^–^^84^ cell lineage diagrams tracing the birth of every cell,^85^^–^^87^ a pan-neuronal atlas strain labeling every neuron non-stochastically,^88^ a signal propagation atlas outlining the functional partners of most head neurons,^89^ single-cell RNAseq transcriptomes,^90^^–^^92^ and the first complete animal genome.^93^^,^^94^ This shared information lends itself to a highly collaborative field capable of high-resolution interrogation of fundamental neurobiological principles. However, many important subcellular structures are smaller than the diffraction limit of light microscopy. Electron microscopy and, more recently, optical super-resolution have been the only methods to collect data about structures such as pre- and postsynaptic specializations, gap junctions, and distinct neurites in a bundle or neuropile.

#### 2.6.1 Adapting expansion microscopy for *C. elegans*

Super-resolution microscopy methods such as STORM, PALM, SR-SIM, or STED have been used to study intact and dissected *C. elegans*,^95^^–^^100^ but the imaging depth of these techniques is insufficient to map the entire depth of the animal. Furthermore, the tough cuticle of *C. elegans* limits antibody penetration and immunohistochemistry important for STORM or STED. The technical difficulty of immunostaining in *C. elegans* likely contributed to the pioneering use of GFP as a reporter in the worm. However, in recent years, the first ExM protocol for *C. elegans* has introduced a cuticle permeabilization technique for immunostaining and expansion^36^ (Fig. 2). Immunostaining allows robust labeling of structures of interest for high-resolution imaging of a large region of interest with reduced constraints from photobleaching of endogenously produced fluorophores.^36^^,^^37^ In this initial proof-of-concept paper, the authors showed that ExM can resolve adjacent synaptic puncta, more than doubling the number of puncta counted pre-expansion.^36^

Several technical challenges remain for a comprehensive optical connectome.^101^ The first challenge is in the complete and continuous segmentation of each neuron, where the identity of each neuron can be maintained across the reach of its neurites. A novel probe has recently been developed expressly for the purpose of dense and continuous membrane labeling in ExM; this azide probe binds to fixed tissue membranes and later is fluorescently labeled via click-chemistry.^102^ The next challenge is in labeling pre- and postsynaptic specializations while retaining contextual information about neuronal identities. Given the mere 20 nm space between opposed membranes of synapses^103^ and the enormous synaptic density, conventional light microscopy cannot parse fluorophore-labeled synapses with the resolution and throughput required to build a connectome. However, with up to 20× physical magnification of samples, ExM could disambiguate individual GFP molecules in the cytosol of neurons;^36^ such resolution has the potential to unpack dense neuronal architecture for optical connectomics.

In the way of protocol optimization and proof of concept, Yu et al.^36^^,^^37^ demonstrated two relevant neuronal features that could be resolved by ExM. One is the disambiguation of individual neurites within a bundle and through defasciculation, as tracking neurites and maintaining neuronal identity is crucial when collecting connectome data. The other feature is the localization of chemical synapses and gap junctions by immunostaining and fluorescence* in situ*. Although synaptic partners can be deduced from membrane proximity, assigning electric and chemical connections, the directions of synaptic connections, and even neurotransmitters, depending on staining specificity, can greatly enrich the connectome data.^104^ These qualitative demonstrations were not driven by an explicit hypothesis but suggest how ExM can provide connectome data.

Thus far, quantitative studies using ExM in *C. elegans* have been mostly cellular rather than neuronal, for example, the ultrastructure organization of P-granules and the dynamics of centrioles. ExM revealed the spatial arrangement of proteins that bind small RNAs in the oocyte P-granule.^43^ P-granules are membrane-less organelles that are detectable by EM, but their protein ultrastructures cannot be discerned because the proteins cannot be differentially labeled for EM. During centriole elimination in oocytes, ExM has been used to reveal the sequence of ultra-structural changes with high spatiotemporal fidelity, where prior analyses have been limited to serial-section EM or immunohistochemistry for either high spatial or temporal resolution, respectively.^105^ Building upon recent advances in the bio-orthogonal click chemistry, multifunctional ExM linkers have been developed to link and label glycans in the ExM gel with high specificity and isotropy, visualizing the distribution of glycans in anatomical context with nanoscale resolution.^106^ Applying click chemistry and its compatibility with ExM adds versatility to label almost limitless molecular targets.

#### 2.6.2 Methods for expansion in *C. elegans* (ExCEL)

Expansion in *C. elegans* (ExCel) has primarily been developed by the Boyden laboratory at MIT.^36^^,^^37^ There are three published protocols for magnifying fixed, whole animals: a standard, an epitope-preserving, and an iterative version (Table 1). The standard protocol allows visualization of fluorescent proteins, which are stable enough to withstand proteinase K digestion. The epitope-preserving protocol uses a gentler digest that allows most endogenous proteins to be labeled by off-the-shelf antibodies. Iterative expansion allows for the highest spatial resolution (∼25  nm). Imaging on a standard confocal microscope provides the following resolutions and limitations.

**Table 1:** Summary of expansion microscopy techniques in *C. elegans*. Table constructed using information from Yu et al.^37^

ExCel variation	Standard ExCEL	Epitope-preserving ExCEL	Iterative ExCEL
Molecular readout	Fluorescent proteins, RNA, DNA, general anatomy	Endogenous proteins	Fluorescent proteins
Linear expansion	3.5× linear expansion	2.8× linear expansion	20× linear expansion
	70-nm resolution	100-nm resolution	25-nm resolution
	High isotropy (1% to 6% error)	Moderate isotropy (8% to 25% error)	High isotropy (1.5% to 4.5% error)
Protocol duration	10 days for fluorophores	18 days	19 days
	14 days for fluorophores and RNAs		
	+1 day for amine-stain for anatomy		
Limitations	RNA, DNA, and anatomy can be revealed through stains or *in situ* hybridization	Proteinase K is replaced with a gentler cuticle-disrupting collagenase and heat-mediated protein digestion. This leads to slightly worse expansion isotropy but allows for readout of ∼70% of endogenous proteins.	Following proteinase K digestion, an oligonucleotide-conjugated antibody is applied to stain target fluorophores. The oligonucleotide transfers and amplifies the stained signal across two rounds of tissue expansion.
	Proteins of interest must be labeled with fluorophores, which are structurally able to withstand the proteinase K digestion that softens tissue for expansion and permeabilizes the worm cuticle for antibody access.		
			Iterative ExCel is more technically demanding and yields a weaker fluorescent signal per voxel than standard ExCel.

## 3 Summary

ExM, in its many varieties, has been used to visualize previously unresolvable subcellular and nano-structures associated with synapses and neuronal processes, in addition to nucleic acids. In the context of neural circuit studies, it was shown to be applicable to resolving fine neural processes, putative synaptic connections between neural populations, fine intra-synaptic structures in both the pre- and post-synaptic specializations, and interactions between glia and neurons. Expansion can now be performed up to 20-fold and be coupled to imaging with a broad variety of fluorescent microscopy technologies, including sub-diffraction microscopy methods, and custom protocols have been published for many organisms. With an appropriate selection of: (1) the staining method, including the decision of whether to stain before expansion or use the protein “decrowding” effect of expansion, which makes targets more accessible and staining more accurate; (2) of the expansion protocol; and (3) of the post-expansion imaging method, it becomes possible to capture the smallest neural features while imaging large neural circuits and adapt the imaging abilities to the requirements of the scientific question being addressed.

The ease of adaptation without the need to obtain new and expensive equipment or alter established staining protocols, the relative simplicity of the protocols, and the unprecedented ability to apply nano-scale imaging to large tissue volumes at high throughput represent a major leap forward toward enabling significant advances in our depth of knowledge about the nervous system. Additional important advances include the automation of sample preparation, imaging, and analysis, facilitating the application of nano-scale imaging and analysis to large animal cohorts.

With a nano-scale imaging technology applicable at high throughput to any genetic model organism in which neural circuit studies are conducted, ExM represents a unique and powerful method for addressing outstanding questions regarding neural circuit structure and function relations. With ExM, we can look at how connectivity patterns and intra-synaptic structure are co-modulated during learning or development to give rise to altered relations between sensory inputs and motor outputs or unravel the structural underpinnings of functional heterogeneity in the performance of sensory-motor transformations and in their adaptation by experience.

## Data Availability

The authors have generated no code or data in this paper.
